# Substrate‐Tight Graphene Transmembrane‐nanofluidic Devices

**DOI:** 10.1002/smll.202407140

**Published:** 2025-02-05

**Authors:** Xiaofang Kang, Buhang Chen, Erik P. van Geest, Wangyang Fu, Jianwei Gao, Luzhao Sun, Zhongfan Liu, Grégory F. Schneider

**Affiliations:** ^1^ Leiden Institute of Chemistry Leiden University Einsteinweg 55 Leiden 2333CC The Netherlands; ^2^ Technology Innovation Center of Graphene Metrology and Standardization for State Market Regulation Beijing Graphene Institute Beijing 100095 P. R. China; ^3^ Key Laboratory of Advanced Materials of Ministry of Education School of Materials Science and Engineering Tsinghua University Shaw Technical Science Building, Haidian District Beijing 100084 P. R. China; ^4^ Center for Nanochemistry Beijing Science and Engineering Center for Nanocarbons Beijing National Laboratory for Molecular Sciences College of Chemistry and Molecular Engineering Peking University Beijing 100871 P. R. China

**Keywords:** adhesion, graphene, ion transport, pyrene, (sub)nanofluidics

## Abstract

Nanopores in 2D membranes like graphene have great potential for applications such as single‐molecule sensing, ion sieving, and harvesting osmotic power. A critical challenge, however, has been to ensure the stability of these nanofluidic transmembrane devices, as the ultrathin graphene membranes tend to delaminate and peel away from their substrates when exposed to aqueous solutions. In this study, it is shown that using a pyrene‐based coating prevents delamination and allows graphene to remain freestanding over a SiN aperture for several days in an electrolyte. The pyrene molecules interact strongly with the graphene through π–π bonding, adhering the graphene to the substrate. Additionally, the pyrene‐based adhesion layer remarkably increases the success rates of the graphene transmembrane devices from 4% to 76.2%. The results underscore the importance of using adhesion layers to enhance the stability of graphene in nanofluidic devices and prolong their operational lifespan. It enables the development of more robust graphene‐based nanofluidic devices for a wide range of applications necessitating free‐standing graphene.

## Graphical abstract

This study demonstrates that the covalent coupling of a pyrene layer to a silicon nitride substrate using silane and peptide chemistry increases the adhesion of graphene to the substrate and prevents the delamination of graphene. 76.2% of the devices with free‐standing graphene are successful devices for ion transport measurement in 0.1 M HCl.

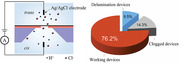

## 1 Introduction

Graphene, with its atomic‐scale thickness, defects, and active sites, has emerged as a highly promising membrane material. Earlier work demonstrated the potential of graphene and its nanopores for characterizing single DNA molecules^[^ ^1^, ^2^, ^3^, ^4^ ^]^ and for efficient ion separation,^[^ ^5^, ^6^ ^]^ both with chemical vapor deposition (CVD)^[^ ^2^, ^3^ ^]^ and exfoliated graphene.^[^ ^6^, ^7^ ^]^ Interestingly, when applying an electrical potential across free‐standing graphene membranes in an electrolyte, a background ionic current is measured,^[^ ^8^ ^]^ originally attributed to ions flowing through pinholes in graphene membranes^[^ ^8^ ^]^ or leakage from the graphene flake edges, even with exfoliated graphene.^[^ ^4^ ^]^

To prevent potential leakage, an efficient strategy involves placing micrometer‐sized washers onto the 2D crystal,^[^ ^6^ ^]^ which involves transferring a photoresist patterned with a micrometer‐sized hole on top of the graphene and baking it further after transfer to ensure good adhesion of the resist polymer to the graphene and to the underlying substrate. This process seals the graphene side edges and clamps them to the substrate, therefore preventing side leakage of ions between the flake and the substrate.

Also crucial to preventing leakage is enhancing the adhesion of graphene with the substrate to minimize water intercalation at the interface between the substrate and the graphene. Thermal annealing (i.e., heating of graphene transferred on the substrate)^[^ ^9^ ^]^ is often used, but the approach does not provide a barrier for water to diffuse between graphene and the substrate which can be hydrophilic.

Another strategy involves the functionalization of the substrate with silane‐based monolayers^[^ ^10^, ^11^ ^]^ to render the substrate hydrophobic and increase the adhesion of graphene. The use of hydrophobic alkyl chains increases the hydrophobic interactions between graphene and the monolayer through van der Waals interactions. Monolayers such as hexamethyldisilazane (HMDS)^[^ ^12^, ^13^ ^]^ and octadecyltrimethoxysilane (OTS)^[^ ^14^ ^]^ are often used in graphene electronic applications because they provide better graphene adhesion to the substrate. Similarly, in the case of a fluidic device, these monolayers could also reduce water intercalation because of their hydrophobicity.

Using pyrene instead of alkyl chains would maximize further the adhesion of graphene to the substrate due to π‐π stacking interactions between the conjugated pyrene and graphene. These pyrene derivatives have been widely used to functionalize graphene field effect transistors^[^ ^15^ ^]^ and were used to anchor additional functional groups, for example using peptide chemistry on N‐hydroxysuccinimide derivatives and pyrene butyric acid moieties.^[^ ^16^, ^17^ ^]^

In this article, we covalently couple a pyrene group to a SiN substrate through a combination of silane and peptide chemistry to enhance the adhesion of free‐standing graphene in electrolytes.^[^ ^18^ ^]^ This layer incorporates a flexible linker made from the amide coupling of the amino propyl side chain of a silane with the butyric acid side chain of the pyrene.^[^ ^17^, ^18^, ^19^ ^]^ The enhanced adhesion between graphene and the SiN substrate suppressed the ion leakage to less than 100 mS cm^−2^ in 0.1 m hydrochloric acid (HCl), similar to the transmembrane conductance reported previously.^[^ ^20^, ^21^, ^22^, ^23^ ^]^ Furthermore, such conductances remained stable over several days of continuous immersion in electrolytes, even under acidic conditions. The pyrene monolayer therefore prevented the delamination of graphene for 76.2% of the devices tested, which now enables the data acquisitions of ion transport for a larger number of free‐standing graphene membrane devices.

## 2 Results and Discussion

### 2.1 Functionalization of the SiN Carrier Chip

**Figure 1:**
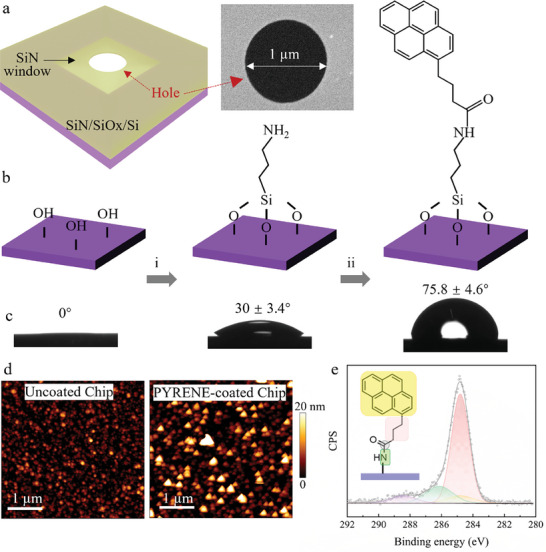
a) Schematic of the chip structure: the purple area represents the silicon substrate covered with a SiN membrane, the central square area (indicated by black arrows) is the suspended SiN window, and the red arrows point to the 1 µm diameter hole in the center. The SEM image of the hole region is shown on the right. b) Schematic representation of the functionalization process: (i) Chips were first treated with oxygen plasma, (ii) and then immersed overnight in a 5 v% APTES solution in ethanol/water (96:4), followed by (iii) pyrene reaction. c) Contact angle images on SiN chip at three stages corresponding to the functionalization steps in (b). d) AFM topography data showing the SiN chip surface morphology before and after functionalization. e) XPS (C1s) spectrum of the SiN chip surface after pyrene functionalization.

A 4 mm × 4 mm silicon chip was fabricated with a 500 µm‐thick silicon base with a 500 nm SiO_2_ layer. A 15 µm × 15 µm window was etched in the center of the SiO_2_ layer, and a 30 nm‐thick silicon nitride (SiN) membrane with a 1 µm aperture was etched on the surface (**Figure** **1**a; Figure S1, Supporting Information). This SiN carrier chip structure is the most common design for 2D membranes and was inspired by the work on solid‐state SiN nanopores.^[^ ^24^ ^]^ The chip surface was then covalently functionalized with a pyrene moiety using a two‐step protocol using 3‐aminopropyl‐triethoxysilane (APTES) and 1‐pyrenebutyric acid (see Methods).

Contact angle (CA) measurements were conducted at various stages of the pyrene functionalization to assess changes in surface hydrophobicity. The pristine chip surface displayed a CA of 55.9 ± 2.5° (five separate chips). Oxygen plasma treatment reduced it to 0° (Figure 1c), indicating increased hydrophilicity due to the introduction of hydroxyl groups (−OH). APTES functionalization slightly increased CA to 30 ± 3.4°, while pyrene functionalization raised it to 75.8 ± 4.6°, confirming enhanced hydrophobicity (Figure 1c). These measurements were obtained from five independent functionalization processes, ensuring robust statistical value.

Atomic force microscopy (AFM) was used to evaluate the surface roughness of the chip before and after pyrene functionalization (Figure 1d). The bare chip showed intrinsic surface roughness of 1.4 ± 0.1 nm, which increased to 2.5 ± 0.8 nm after pyrene functionalization. X‐ray photoelectron spectroscopy (XPS) analysis verified the integration of nitrogen after APTES treatment and the presence of sp^2^‐bonded carbon atoms consequent to pyrene functionalization on the chip surface. The N1s peak marked APTES modification (Figure S2, Supporting Information), and Figure 1e details the XPS spectrum of the pyrene‐functionalized chip, where a distinct peak in the carbon 1s region is evident. Analysis of the binding energy spectrum reveals discrete components corresponding to various carbon bonds intrinsic to the pyrene molecule (inset). The peak exhibiting the highest intensity, positioned at ≈284.5 eV, aligns with the C─C bonds in the aromatic ring characteristic of pyrene. Additional peaks, observed at higher binding energies, are associated with carbon atoms in various chemical environments, including C─O, C═O, and C─N functionalities, indicative of the functional groups attached to the pyrene. The deconvolution of these peaks underscores the substantial presence of sp^2^ hybridized carbon from pyrene, thus confirming the successful functionalization of the chip surface with pyrene entities.

We also used UV–Vis spectroscopy to quantify the amount of pyrene on the chip surface. Since UV–Vis cannot be performed on a fluidic chip due to its lack of light transmittance, we used a quartz substrate as a substitute to determine the pyrene density. We assumed that the surface properties of the chip and of the quartz substrate were comparable, as the chip's surface is only covered by a thin layer of SiN (on the nanometer scale). By measuring the pyrene density on four independently pyrene functionalized quartz substrates, we determined an average pyrene surface density of 0.251 ± 0.039 nmol cm^−^^2^ (0.723 ± 0.028 g m^−^^2^) (Figure S3, Supporting Information), which is comparable to the value reported by Miskin et al.^[^ ^18^ ^]^

### 2.2 Ion Transport Measurements of Pyrene‐Functionalized Chips Without Graphene

**Figure 2:**
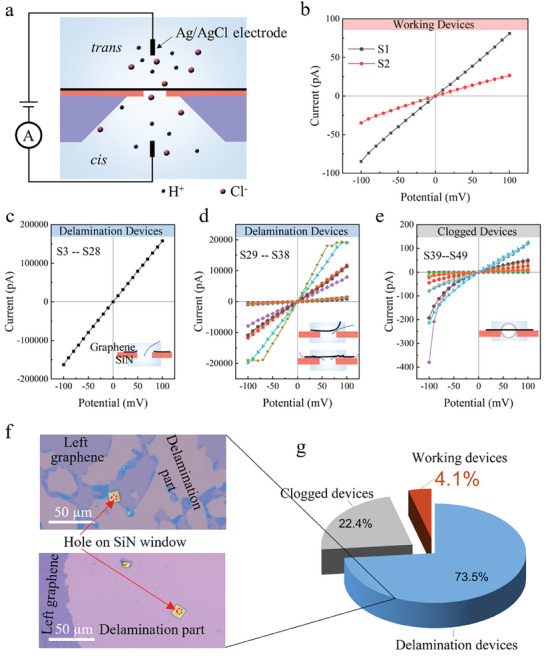
Ion transport measurements in graphene fluidic devices without a pyrene layer. a) Schematic of the ionic current measurement setup using a patch clamp: A microchip with a graphene‐covered hole is positioned in a flow cell with two 0.1 m HCl reservoirs. The ionic current was measured by applying a bias between two saturated Ag/AgCl electrodes immersed on both sides of the micro‐hole. The *I*–*V* curves illustrate various observed scenarios: b) successful devices with expected protonic ionic current, c) complete damage to suspended graphene, resulting in conductance levels comparable to those of the blank chip, d) varying degrees of delamination in the graphene layer, leading to higher proton conductance than typical values reported for CVD graphene, e) issues with improper wetting and bubble formation in devices, causing highly asymmetric *I*–*V* curves. f) Optical image after ion transport measurement. g) A summary statistic chart illustrating the outcomes of all 49 tested devices.

A typical ion transport device consists of a SiN carrier chip with a hole mounted in a polymethyl methacrylate flow cell. A 1:1 mixture of ultrapure water and ethanol is introduced to wet the microflow channel and chip surface. Ion movement is induced by applying a voltage across the *cis* and *trans* chamber using Ag/AgCl electrodes, each containing a 0.1 m HCl electrolyte (**Figure** **2**a).

To verify the process of pyrene functionalization, the suspended SiN membrane was inspected for possible damage or micropore clogging. The chip surface was randomly examined before transferring graphene onto the chip (Figure S4a, Supporting Information). For transconductance tests on bare chips, bias voltages ranging from −100 to 100 mV were applied in increments of 10 mV, and the resulting currents were recorded. The current‐voltage (*I*–*V*) curves were obtained by averaging the ion current time series at each voltage step. As shown in Figure S4b (Supporting Information), the *I*–*V* curves for the seven blank chips exhibit linear behavior, indicating the presence of ohmic resistance. The conductivity was determined using the equation *g = I/V*. The conductance values presented in Figure S4c (Supporting Information) show that the pore conductance (g_chip_) is 2544 ± 139 nS (across seven chips) in a 0.1 m HCl solution. The pore diameter *D* was then determined by Equation (1).^[^ ^25^ ^]^

(1) $$g=\sigma {\left(\frac{4l}{\pi D^{2}}+\frac{1}{D}\right)}^{-1}$$

Here, the bulk conductivity *σ* measured using a conductivity meter is 3.7 S m^−1^ for 0.1 m HCl. The pore length *l* corresponds to the thickness of the SiN membrane (30 nm), and *D* is the calculated pore diameter. As shown in Figure S4c (Supporting Information), the calculated pore diameter is ≈1 µm, and the AFM image of the surrounding area (Figure S5, Supporting Information) confirms the presence of a through‐aperture in the chip used for further graphene‐based sub‐nanofluidic devices.

### 2.3 Ion Transport Measurements in Graphene Devices Without a Pyrene Layer

To achieve high‐yield graphene‐based sub‐nanofluidic devices, we chose high‐quality chemical vapor deposition (CVD) graphene^[^ ^26^ ^]^ instead of exfoliated graphene, it offers centimeter‐scale sheets that are easier to manipulate and more scalable for production. The CVD graphene used in our work had domain sizes up to A3‐size (≈0.42 × 0.3 m^2^),^[^ ^26^ ^]^ effectively avoiding the number of interdomain boundaries within the free‐standing area. Figure S6 (Supporting Information) presents a measured in‐plane sheet resistance of 616 ± 78 Ω sq^−1^ across a 5 cm × 5 cm region, as illustrated in the inset, which provides a mapping of sheet resistance, where fluctuations in sheet resistance values of less than 13%, demonstrating the integrity and uniformity of CVD graphene films at the scale above centimeters. Additionally, Figure S7a,b (Supporting Information) displays the mapping of the I_D_/I_G_ and I_2D_/I_G_ ratios over a 60 µm × 60 µm area, indicating a nearly negligible defect density and uniformity of the single‐layer graphene. Raman spectra obtained from five different spots across a centimeter‐scale area (Figure S7c, Supporting Information) showed no significant defective peaks, further supporting the graphene's high quality and uniformity consistency. The graphene was transferred onto the SiN chip with a 1 µm hole (Figure 2a) using a poly(methyl methacrylate) (PMMA)‐assisted transfer method (see Methods for details).

We evaluated device performance with and without the pyrene layer. Initially, devices were tested without the pyrene layer. The area‐normalized conductance (g_areal_) was derived as g_areal_ = *g/A*, where A represents the suspended graphene area, calculated for a diameter of 1 µm. The area‐normalized proton conductance values for the two devices depicted in Figure 2b are 103.2 and 33.1 mS cm^−2^, which is consistent with previously reported conductance ranges of 4–100 mS cm^−2^ in the literature.^[^ ^20^, ^23^, ^24^, ^25^, ^26^, ^27^ ^]^ We refer to devices that exhibit the intrinsic transmembrane properties of graphene as “working devices” (Figure 2g). Recent research has focused on understanding the mechanisms of proton conduction in graphene, identifying several potential pathways: (1) proton transport through atomic‐scale defects in the graphene lattice,^[^ ^27^, ^28^, ^29^ ^]^ (2) solution‐mediated hydrogenation of graphene,^[^ ^30^, ^31^ ^]^ and (3) the wrinkles and nanoripples in suspended graphene, lowering the activation energy for proton translocation.^[^ ^32^ ^]^

However, Figure 2c reveals that 49% of the devices (24 out of 49) exhibited conductance levels comparable to the bare chips, indicating that suspended graphene rapidly delaminates from the substrate when exposed to a strong acid electrolyte environment. This phenomenon can be attributed to the weak adhesion energy between the graphene and the underlying substrate, which is insufficient to maintain the graphene adhering to the substrate in the electrolyte. Furthermore, 21% of the devices (Figure 2d) showed proton conductance higher than the typical values reported for CVD graphene.^[^ ^20^, ^21^, ^22^, ^23^ ^]^ Notably, it can be observed from Figure S8 (Supporting Information) that the conductance rises as the number of electrolyte substitutions in S29 increases, suggesting that graphene is gradually delaminating from the substrate.

Additionally, 22% of the samples exhibited highly asymmetric *I*–*V* curves, classified as clogged devices. This issue could originate from inadequate wetting of the micro flow channel or graphene membrane,^[^ ^33^, ^34^ ^]^ despite using a 50% ethanol: 50% water solution for pre‐wetting. Other potential causes include the introduction of air bubbles during solution injection or the presence of polymer or hydrocarbon‐related contamination.^[^ ^35^, ^36^ ^]^

Unfortunately, only 4% of devices demonstrated the intrinsic transmembrane properties of the graphene, indicating an extremely low device yield. In 73.5% of devices, as shown in Figure 2f, optical imaging revealed significant delamination after ion transport measurements. This extensive delamination, affecting 73.5% of the devices, clearly demonstrates weak graphene adhesion to the substrate, which directly contributes to the low yield.

### 2.4 Ion Transport Measurements in Graphene Devices with a Pyrene Layer

**Figure 3:**
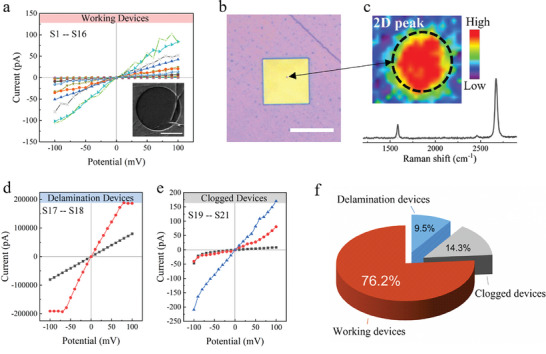
Conductances of free‐standing graphene membranes on pyrene‐functionalized chips. a) *I*–*V* curves from intact devices exhibit conductance below 100 mS cm^−2^. Inset: SEM image of a representative device (Sample 1–16). Scale bar: 500 nm. b) Optical image of a pyrene‐functionalized chip with graphene after ion transport measurement, the regions of suspended graphene are indicated by black dots. Scale bar: 10 µm. c) Raman spectroscopy 2D peak mapping and associated spectral data confirm the presence and quality of the suspended graphene corresponding to image (b). d) *I*–*V* curves of devices with high leakage currents (Sample 17,18). e) *I*–*V* curves of clogged devices (Sample 19–21). f) Aggregated statistical analysis of all tested pyrene‐functionalized graphene devices.

To evaluate the impact of the pyrene layer, we transferred the single crystalline CVD graphene onto the chip and conducted ionic current measurements. These results were systematically compared with those obtained from devices without the pyrene layer. As illustrated in **Figure** **3**a, 76.2% of devices exhibited linear *I*–*V* curves and a transconductance of 1–100 mS cm^−2^ (61 ± 46 mS cm^−2^) in a 0.1 m HCl electrolyte, suggesting that there is no delamination occurring. To the best of our knowledge, the conductance variations among devices are attributed to the inevitable introduction of wrinkles/ripples during graphene transfer, which reduces the energy barrier for proton transport through the graphene membrane.^[^ ^32^, ^37^ ^]^ The uncontrolled transfer process leads to varying wrinkles/ripple densities in the suspended graphene, resulting in different proton penetration and conductance deviations among devices. SEM images (the inset of Figure 3a) confirmed complete graphene coverage over the pore area, supporting this hypothesis.

The optical image in Figure 3b reveals a uniform optical contrast, further indicating that the graphene remains intact and has not undergone delamination, including the free‐standing area. Raman spectroscopy further validated the graphene coverage, as evidenced by sharp 2D and G peaks without the emergence of D peaks, suggesting no induced mechanical damages (Figure 3c). In the 2D peak mapping, the robust fluorescence signal emitted by SiN may obscure the graphene signal in the SiN‐supported area. However, the strong 2D signal of suspended graphene remains clearly recognizable across the entire freestanding area.

A few devices (Figure 3d) showed high leakage current, and SEM images (Figure S9, Supporting Information) revealed localized graphene damage potentially arising from the transfer process. The comparable instances of clogged devices between pyrene‐functionalized and uncoated devices suggest that the entire pyrene functionalization procedure does not introduce chip contamination. Although annealing was considered a potential cleaning method to address graphene surface contamination, it was ultimately discarded due to concerns about potential damage to the pyrene layer at high temperatures. Despite these occurrences, the integration of the pyrene layer significantly improved the success rate of device production, increasing it from 4.1% to 76.2%.

**Figure 4:**
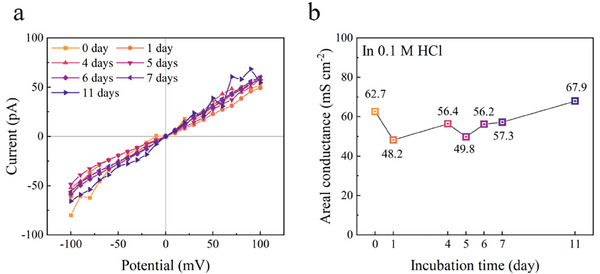
Evaluating the stability of graphene devices with pyrene‐functionalized chips in an acidic environment. a) *I*–*V* curves at different incubation times in 0.1 m HCl. d) Evolution of areal conductance over the incubation period.

To assess the mechanical stability of graphene under acidic conditions, we randomly selected one device from the “16 working devices” for comprehensive analysis. The conductance (*g*) of this device was continuously monitored during immersion in 0.1 m HCl. A stepwise incubation method was employed to systematically track the ionic current of the devices over time. As shown in the I‐V curves in **Figure** **4**a, the initial current values and those after 11 days of immersion remained within the same order of magnitude, with the areal conductance maintained at ≈60 mS cm⁻^2^ (Figure 4b), demonstrating the high stability of graphene membrane in acid aqueous conditions. The devices with pyrene‐functionalized chips maintained consistent conductance throughout the incubation period, indicating the robustness of the graphene membrane. Furthermore, SEM and Raman spectra from the 1 µm pore region, collected after 11 days of testing (Figure S10, Supporting Information), reveal that the graphene maintained its characteristic monolayer Raman signals and completely covered the pore region, further confirming its structural integrity.

## 3 Conclusion

In this study, we have developed a novel fabrication process to protect CVD graphene from damage during ionic transport measurements in graphene‐based sub‐nanofluidic devices. By incorporating a covalently bonded pyrene layer, we successfully functionalized the chip surface, introducing nitrogen and sp^2^ carbon, as confirmed by XPS. The adhesive layer enhances surface hydrophobicity and strong π–π interactions between the graphene and the substrate, significantly reducing leakage at the graphene/SiN interface. Consequently, the success rate of graphene nanofluidic devices increased to ≈76.2%, and the transmembrane conductance of graphene remained stable even in strongly acidic environments.

This surface modification technique represents a significant advancement in the fabrication of diverse graphene‐based devices. By addressing the critical issue of graphene damage during ionic transport measurements, our approach enables the development of more robust and reliable graphene‐based sub‐nanofluidic devices. We anticipate that this technique will be complementary to other approaches or devices used for ion transport through membranes, facilitating the exploration of novel applications and furthering our understanding of ionic transport phenomena at the nanoscale.

## 4 Experimental Section

### Materials

Large‐area, high‐quality monocrystalline graphene, was provided by our collaborators at the Beijing Graphene Institute, and the growth process was described in the “*Single‐crystalline graphene growth*” section below. Ultrapure water, with a resistivity of 18.2 MΩ.cm, was obtained from a Millipore Milli‐Q gradient A10 system. All gases used in this study were supplied by Linde Gas (5.0). Custom‐made silicon nitride (SiN) carrier chips were fabricated on a full wafer, with processing information provided in Figure S1 (Supporting Information). All chemicals, including ammonium persulfate (APS), 37% hydrochloric acid (HCl), acetone, isopropyl alcohol (IPA), ethanol, N, N‐dimethylformamide (DMF), 1‐[bis(dimethylamino)methylene]‐1H‐1,2,3‐triazolo[4,5‐b]pyridinium 3‐oxid hexafluorophosphate (HATU), 3‐aminopropyl‐triethoxysilane (APTES), 1‐pyrenebutyric acid (PBA), and triethylamine (NEt₃), were purchased from Sigma–Aldrich, and used as received without further purification. Poly(methyl methacrylate) (PMMA) with a 6% concentration in anisole (AR‐P 662.06) was purchased from Allresist GmbH.

### Single‐Crystalline Graphene Growth

High‐quality single‐crystalline graphene film was synthesized on Cu(111) foils via chemical vapor deposition (CVD) method.^[^ ^26^ ^]^ The commercial polycrystalline Cu foils were placed in a homemade low‐pressure CVD system, equipped with three heating zones and a 6‐inch quartz tube. An asynchronous heating process was conducted to create a suitable temperature gradient across the Cu foils, thereby facilitating the growth of abnormal grain and leading to the formation of large‐area single crystal.^[^ ^38^ ^]^ The sample was annealed under a 1000 sccm flow of Ar (40 min) and a 1000 sccm flow of H_2_ (20 min). Subsequently, graphene was grown on the resulting single crystal Cu(111) foil for 1 h using a gas mixture of Ar (500 sccm), H_2_ (500 sccm), CH_4_ (1.6 sccm) and trace amounts of O_2_ (0.4 sccm). The chamber pressure was maintained at 1000 Pa throughout the CVD process.

### Single‐Crystalline CVD Graphene Sub‐Nanofluidics Preparation

The so‐called PMMA‐assisted transfer method was used for device preparation. In this process, the PMMA was spin‐coated (4000 rmp for 60 sec) on the top side of graphene on the copper foil using a POLOS SPIN150i tabletop spin coater and then placed on a hot plate at 80 °C for 2 min to ensure complete drying of the PMMA. With the PMMA side facing down, the exposed backside of the graphene was treated with oxygen plasma using a capacitively coupled plasma (CCP) system from Diener electronics at 100 W (50%), 0.30 mbar for 2 min. Subsequently, the copper foil, with the PMMA side up, was floated on 0.1 m APS solution and etched until the copper was completely dissolved. The PMMA/graphene film was then transferred in ultrapure water three times to ensure thorough rinsing and removal of any residual APS solution. Next, the PMMA/graphene film was bottom‐fished onto a SiN carrier chip with a pre‐fabricated hole. The chip was then partially dipped into the IPA using tweezers to remove the water trapped between graphene and the substrate, and left at room temperature to allow gentle evaporation. After complete water evaporation, the chip was heated at 80 °C for 30 min. Finally, the PMMA layer was removed by immersing it in acetone for 10 min, followed by rinsing with acetone, IPA, and ethanol and drying with an N_2_ gun.

### Pyrene Functionalization

This pyrene functionalizationprocess for SiN chip is based on a pyrene functionalization method developed in our group for Si/SiO2 wafer^[^ ^17^ ^]^ and involved two steps. Initially, the chip was sequentially rinsed with acetone, IPA, and ethanol, and then blow‐dried with a nitrogen gun. The chip surface was then treated with oxygen plasma using a capacitively coupled plasma (CCP) system from Diener electronics at 100 W (50%), 0.30 mbar for 2 min. Immediately after plasma treatment, the chip was immersed overnight in a 5 solution of APTES (1.5 mL) in 96% ethanol (30 mL). The next day, the chip was taken from the solution, rinsed with acetone, ultrapure water, IPA, and blow‐dried with nitrogen. For pyrene reaction, a solution of HATU (255 mg, 22 mm) and PBA (129 mg, 15 mm) in DMF was prepared and activated for 10 min. 12 droplets of NEt_3_ were added to this mixture (30 mL DMF), and the freshly APTES‐functionalized chip was immersed in the solution for three days. Finally, the chip was rinsed with ethanol and blow‐dried with nitrogen.

### Characterization and Measurement

Raman spectroscopy was conducted using a WITEC alpha500 R Confocal Raman Imaging system with a 532 nm laser wavelength. The laser power was controlled below 2 mW to mitigate potential heating effects. Spectra were collected using a 100 × objective lens, and spatial mapping was conducted over a selected area of 2 µm × 2 µm, including the entire free‐standing CVD graphene. The mapping involved scanning in both the horizontal and vertical directions, with each row and column consisting of 20 points. Each data point was measured with an integration time of 0.50 s. Measurements were carried out in air at room temperature, and data analysis was conducted using WITEC GmbH software and OriginPro (V9.1) software.

Contact angle measurement was performed using a Ramé‐Hart 250 goniometer (Netcong, NJ) and recorded using DROPimage advanced v 2.8 software at room temperature. A 2 µL droplet of Milli‐Q water was vertically placed on the substrate surface using a micropipette. The contact angle was measured within 5 s to minimize evaporation effects and ensure precise readings.

Optical images were obtained with a Leica DM 2700 m Brightfield microscope containing a Leica MC 120 HD camera.

Scanning electron microscopy (SEM) was conducted using a JEOL SEM 6400 microscope to visually check the graphene coverage and identify graphene‐damaged areas with high leakage current devices. Imaging was conducted under high vacuum conditions with an accelerating voltage of 10 kV, a current of 0.1 nA, and a working distance of ≈10 mm.

Atomic force microscopy (AFM) was performed using a JPK NanoWizard Ultra Speed machine and processed with JPK SPM Data Processing software. The measurements employed a silicon probe (OPUS, 160AC‐NA) with a nominal resonance frequency of 300 kHz and a spring constant of 20 N m^−1^. Imaging was conducted in AC (tapping) mode at room temperature in air, with a resolution of 512 × 512 pixels. The images were then processed using JPK SPM Data Processing software.

X‐ray photoelectron spectroscopy (XPS) was performed using a Thermo Fisher ESCALB Xi+ instrument with 150 W A1 Kα X‐ray source. The spectra were analyzed and fitted using CasaXPS software with Gaussian (70%)‐Lorentzian (30%) (GL(30)) line shape and Shirley background.

UV–Vis absorption spectra were measured using a PerkinElmer Lambda 35 spectrophotometer, with a wavelength range of 190–1100 nm and a step size of 1 nm, using 1 cm quartz cuvettes. Standard solutions were prepared by dissolving 1‐pyrene butyric acid in 96% v/v ethanol, with baseline calibration using an empty quartz cuvette. Sample measurements were conducted on pyrene‐modified quartz slides, calibrated with the same blank quartz slide. All measurements were performed at room temperature.

The conductivity of the electrolytes was measured using an Edge EC, 230 V (HI2003‐02) meter. Before each measurement, the meter was calibrated with conductivity standards of 84 and 12880 µS cm^−1^ to ensure accuracy.

All the ionic transport measurements were carried out using an Axopatch 200B amplifier coupled with a Digitizer 1550 (both from Molecular Devices). All potentials were referenced to a saturated Ag/AgCl electrode. Measurements were conducted at room temperature in a Faraday cage on a vibration isolation table to minimize noise.

## Conflict of Interest

The authors declare that a patent has been filed on the content of reference.^[^ ^17^ ^]^

## Supporting information



Supporting Information

## Data Availability

The data that support the findings of this study are available from the corresponding author upon reasonable request.

## References

[1] S. J. Heerema , C. Dekker , Nat. Nanotechnol. 2016, 11, 127. 26839258 10.1038/nnano.2015.307

[2] S. Garaj , W. Hubbard , A. Reina , J. Kong , D. Branton , J. A. Golovchenko , Nature 2010, 467, 190. 20720538 10.1038/nature09379 PMC2956266

[3] C. A. Merchant , K. Healy , M. Wanunu , V. Ray , N. Peterman , J. Bartel , M. D. Fischbein , K. Venta , Z. Luo , A. T. Johnson , M. Drndic , Nano Lett. 2010, 10, 2915. 20698604 10.1021/nl101046t

[4] G. F. Schneider , S. W. Kowalczyk , V. E. Calado , G. Pandraud , H. W. Zandbergen , L. M. Vandersypen , C. Dekker , Nano Lett. 2010, 10, 3163. 20608744 10.1021/nl102069z

[5] R. C. Rollings , A. T. Kuan , J. A. Golovchenko , Nat. Commun. 2016, 7, 11408. 27102837 10.1038/ncomms11408 PMC4844701

[6] L. Mogg , S. Zhang , G. P. Hao , K. Gopinadhan , D. Barry , B. L. Liu , H. M. Cheng , A. K. Geim , M. Lozada‐Hidalgo , Nat. Commun. 2019, 10, 4243. 31534140 10.1038/s41467-019-12314-2 PMC6751181

[7] S. Hu , M. Lozada‐Hidalgo , F. C. Wang , A. Mishchenko , F. Schedin , R. R. Nair , E. W. Hill , D. W. Boukhvalov , M. I. Katsnelson , R. A. Dryfe , I. V. Grigorieva , H. A. Wu , A. K. Geim , Nature 2014, 516, 227. 25470058 10.1038/nature14015

[8] J. L. Achtyl , R. R. Unocic , L. Xu , Y. Cai , M. Raju , W. Zhang , R. L. Sacci , I. V. Vlassiouk , P. F. Fulvio , P. Ganesh , D. J. Wesolowski , S. Dai , A. C. van Duin , M. Neurock , F. M. Geiger , Nat. Commun. 2015, 6, 6539. 25781149 10.1038/ncomms7539 PMC4382684

[9] Y. T. Megra , S. Lim , T. Lim , S. R. Na , J. W. Suk , Appl. Surf. Sci. 2021, 570, 151243.

[10] F. Schreiber , Prog. Surf. Sci. 2000, 65, 151.

[11] A. Ulman , Chem. Rev. 1996, 96, 1533. 11848802 10.1021/cr9502357

[12] M. Thakur , N. Cai , M. Zhang , Y. Teng , A. Chernev , M. Tripathi , Y. Zhao , M. Macha , F. Elharouni , M. Lihter , L. Wen , A. Kis , A. Radenovic , npj 2D Mater. Appl. 2023, 7, 11. 38665480 10.1038/s41699-023-00373-5 PMC11041726

[13] S. Ramadan , Y. Zhang , D. K. H. Tsang , O. Shaforost , L. Xu , R. Bower , I. E. Dunlop , P. K. Petrov , N. Klein , ACS Omega 2021, 6, 4767. 33644584 10.1021/acsomega.0c05631 PMC7905810

[14] S. Y. Chen , P. H. Ho , R. J. Shiue , C. W. Chen , W. H. Wang , Nano Lett. 2012, 12, 964. 22224857 10.1021/nl204036d

[15] C. Dai , Y. Liu , D. Wei , Chem. Rev. 2022, 122, 10319. 35412802 10.1021/acs.chemrev.1c00924

[16] K. Thodkar , P. A. Cazade , F. Bergmann , E. Lopez‐Calle , D. Thompson , D. Heindl , ACS Appl. Mater. Interfaces 2021, 13, 9134. 33573369 10.1021/acsami.0c18485

[17] E. P. van Geest , B. Can , M. Makurat , C. Maheu , H. Sezen , M. D. Barnes , D. Bijl , M. Buscema , S. Shankar , D. J. Wehenkel , R. van Rijn , J. P. Hofmann , J. M. van Ruitenbeek , G. F. Schneider , Preprint, 2025, https://arxiv.org/abs/2501.12963. 10.1039/d6cc01037g PMC13330647 42397186

[18] M. Z. Miskin , C. Sun , I. Cohen , W. R. Dichtel , P. L. McEuen , Nano Lett. 2018, 18, 449. 29272587 10.1021/acs.nanolett.7b04370

[19] Y. Ueno , K. Dendo , Y. Homma , K. Furukawa , Sensors and Materials 2019, 31, 1157.

[20] H. Qi , Z. Li , Y. Tao , W. Zhao , K. Lin , Z. Ni , C. Jin , Y. Zhang , K. Bi , Y. Chen , Nanoscale 2018, 10, 5350. 29509202 10.1039/c8nr00050f

[21] P. Chaturvedi , N. K. Moehring , P. Cheng , I. Vlassiouk , M. S. H. Boutilier , P. R. Kidambi , J. Mater. Chem. A 2022, 10, 19797.

[22] M. I. Walker , P. Braeuninger‐Weimer , R. S. Weatherup , S. Hofmann , U. F. Keyser , Appl. Phys. Lett. 2015, 107, 213204.

[23] P. Chaturvedi , I. V. Vlassiouk , D. A. Cullen , A. J. Rondinone , N. V. Lavrik , S. N. Smirnov , ACS Nano 2019, 13, 12109. 31592639 10.1021/acsnano.9b06505

[24] C. Dekker , Nat. Nanotechnol. 2007, 2, 209. 18654264 10.1038/nnano.2007.27

[25] S. W. Kowalczyk , A. Y. Grosberg , Y. Rabin , C. Dekker , Nanotechnology 2011, 22, 315101. 21730759 10.1088/0957-4484/22/31/315101

[26] L. Sun , B. Chen , W. Wang , Y. Li , X. Zeng , H. Liu , Y. Liang , Z. Zhao , A. Cai , R. Zhang , Y. Zhu , Y. Wang , Y. Song , Q. Ding , X. Gao , H. Peng , Z. Li , L. Lin , Z. Liu , ACS Nano 2022, 16, 285. 34965103 10.1021/acsnano.1c06285

[27] C. L. Bentley , M. Kang , S. Bukola , S. E. Creager , P. R. Unwin , ACS Nano 2022, 16, 5233. 35286810 10.1021/acsnano.1c05872 PMC9047657

[28] Y. An , A. F. Oliveira , T. Brumme , A. Kuc , T. Heine , Adv. Mater. 2020, 32, 2002442. 10.1002/adma.202002442 32743870

[29] E. Griffin , L. Mogg , G. P. Hao , G. Kalon , C. Bacaksiz , G. Lopez‐Polin , T. Y. Zhou , V. Guarochico , J. Cai , C. Neumann , A. Winter , M. Mohn , J. H. Lee , J. Lin , U. Kaiser , I. V. Grigorieva , K. Suenaga , B. Ozyilmaz , H. M. Cheng , W. Ren , A. Turchanin , F. M. Peeters , A. K. Geim , M. Lozada‐Hidalgo , ACS Nano 2020, 14, 7280. 32427466 10.1021/acsnano.0c02496

[30] M. Bartolomei , M. I. Hernández , J. Campos‐Martínez , R. Hernández‐Lamoneda , Carbon 2019, 144, 724.

[31] Y. Feng , J. Chen , W. Fang , E. G. Wang , A. Michaelides , X. Z. Li , J. Phys. Chem. Lett. 2017, 8, 6009. 29185752 10.1021/acs.jpclett.7b02820

[32] O. J. Wahab , E. Daviddi , B. Xin , P. Z. Sun , E. Griffin , A. W. Colburn , D. Barry , M. Yagmurcukardes , F. M. Peeters , A. K. Geim , M. Lozada‐Hidalgo , P. R. Unwin , Nature 2023, 620, 782. 37612394 10.1038/s41586-023-06247-6 PMC10447238

[33] J. W. Polster , F. Aydin , J. P. de Souza , M. Z. Bazant , T. A. Pham , Z. S. Siwy , J. Am. Chem. Soc. 2022, 144, 11693. 35729706 10.1021/jacs.2c03436 PMC9264351

[34] X. Kang , W. Fu , G. F. Schneider , Carbon 2025, 234.

[35] Y. Wu , Y. Qian , B. Niu , J. Chen , X. He , L. Yang , X. Y. Kong , Y. Zhao , X. Lin , T. Zhou , L. Jiang , L. Wen , Small 2021, 17, 2101099. 10.1002/smll.202101099 34121315

[36] P. Y. Apel , I. V. Blonskaya , O. L. Orelovitch , P. Ramirez , B. A. Sartowska , Nanotechnology 2011, 22, 175302. 21411914 10.1088/0957-4484/22/17/175302

[37] P. Z. Sun , W. Q. Xiong , A. Bera , I. Timokhin , Z. F. Wu , A. Mishchenko , M. C. Sellers , B. L. Liu , H. M. Cheng , E. Janzen , J. H. Edgar , I. V. Grigorieva , S. J. Yuan , A. K. Geim , Proc Natl Acad Sci U S A 2023, 120, 2300481120. 10.1073/pnas.2300481120 PMC10041176 36913585

[38] Y. Li , L. Sun , Z. Chang , H. Liu , Y. Wang , Y. Liang , B. Chen , Q. Ding , Z. Zhao , R. Wang , Y. Wei , H. Peng , L. Lin , Z. Liu , Adv. Mater. 2020, 32, 2002034. 10.1002/adma.202002034 32529704

