# Supplementary material for: Substrate‐Tight Graphene Transmembrane‐nanofluidic Devices
Source: Small. 2025 Feb 5;22(25):2407140. doi: 10.1002/smll.202407140 (PMC13137235; doi:10.1002/smll.202407140)
Supplement: Supplementary file 1 — Supporting Information [file SMLL-22-2407140-s001.docx]

*Supporting information for*

Substrate-tight graphene transmembrane-nanofluidic devices

Xiaofang Kang^1^, Buhang Chen^2^, Erik P. van Geest^1^, Wangyang Fu^3^*, Jianwei Gao^1^, Luzhao Sun^2,4^_,_ Zhongfan Liu^2,4^, Grégory F. Schneider^1^*

*^1^Leiden Institute of Chemistry, Leiden University, Einsteinweg 55, 2333CC Leiden, The Netherlands*

*^2^ Technology Innovation Center of Graphene Metrology and Standardization for State Market Regulation, Beijing Graphene Institute, Beijing, 100095, People’s Republic of China*

*^3^ Key Laboratory of Advanced Materials of Ministry of Education, School of Materials Science and Engineering, Tsinghua University, Shaw Technical Science Building, Beijing, Haidian District 100084, People’s Republic of China*

*^4^ Center for Nanochemistry, Beijing Science and Engineering Center for Nanocarbons, Beijing National Laboratory for Molecular Sciences, College of Chemistry and Molecular Engineering, Peking University, Beijing 100871, People’s Republic of China*

* To whom correspondence should be addressed:

[fwy2018@mail.tsinghua.edu.cn](mailto:fwy2018@mail.tsinghua.edu.cn)

[g.f.schneider@chem.leidenuniv.nl](mailto:g.f.schneider@chem.leidenuniv.nl)

**
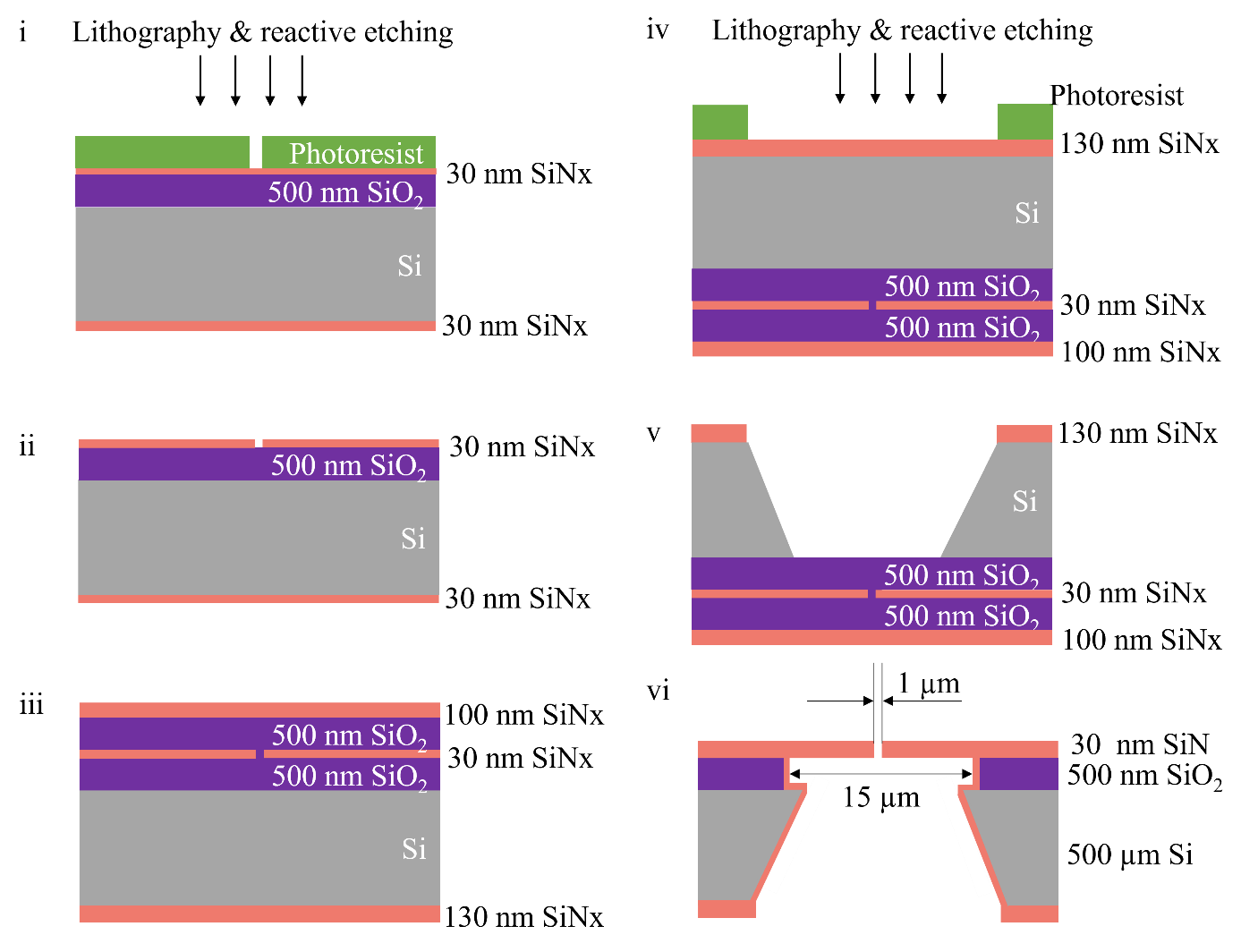
**

**Figure S1. Lithographic steps used in the fabrication of the SiNx chip**. (i) After the PECVD oxide and the LPCVD membrane nitride layer are deposited, lithography is done to expose a photo-sensitive resist layer with a stepper reticle. The reticle is designed in such a way that the resist is illuminated only with a 1 µm in diameter disk. (ii) After development, the exposed resist is dissolved. The nitride layer is open and is etched away by RIE etching. (iii) After stripping the resist there are two sacrificial layers deposited on top – a PECVD oxide layer and an LPCVD nitride layer – to protect the LPCVD membrane nitride layer for step (v). (iv)On the backside of the wafer, there is again a lithographical step done by using a second reticle. By RIE etching, the nitride is etched away. This forms the window for further KOH etching. (v) In a KOH solution, the Si substrate is etched selectively to the lattice of the Si. KOH is etching <1,0,0> planes 100 times faster than <1.1.1> planes. The KOH lands on the backside of the first PECVD oxide layer. (vi)At the frontside, the last sacrificial LPCVD nitride is stripped by RIE etching. The PECVD oxide layers on both sides of the LPCVD membrane nitride layer are etched in a buffered oxide etch solution.


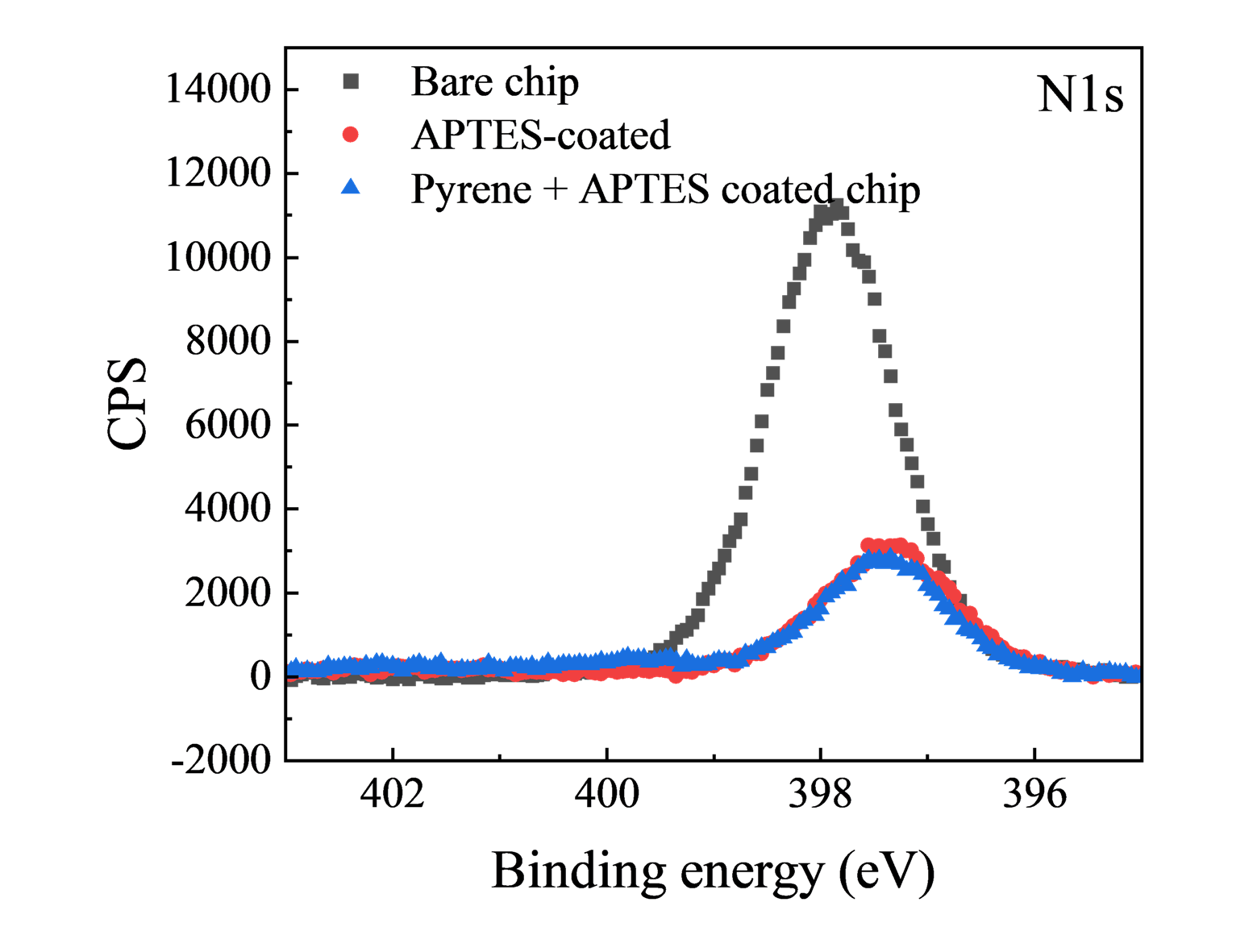


**Figure S2.** Nitrogen (N1s) content of the bare SiN chip, and after APTES, and pyrene-functionalization. CPS = counts per second.


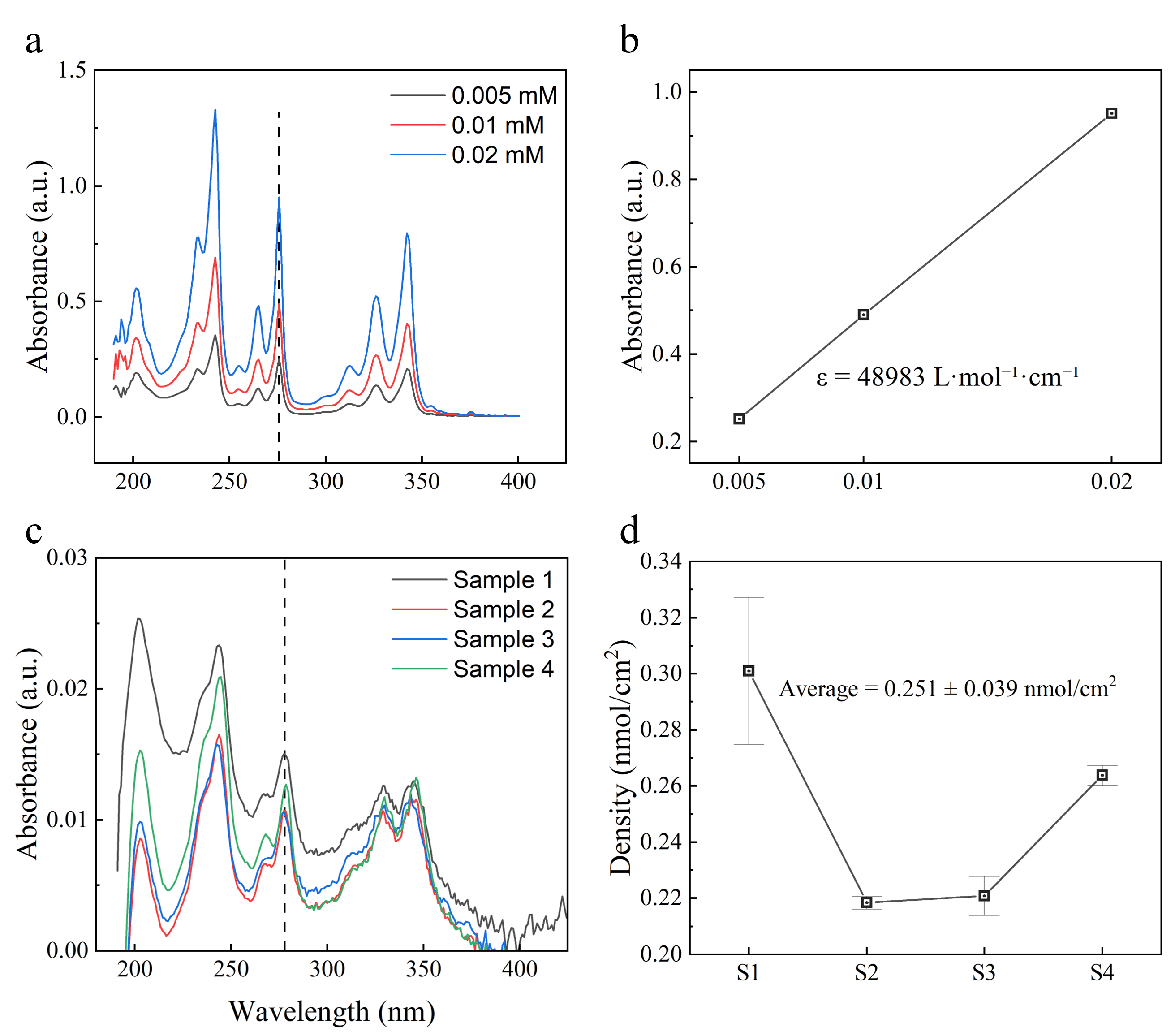


**Figure S3.** UV-vis absorption spectra of 1- pyrene butyric acid at three different concentrations, highlighting absorption peak. (b) The linear correlation between absorbance at 280 nm and pyrene concentration demonstrates adherence to the Beer-Lambert law. (c) UV-vis absorption spectra of four independently pyrene-functionalized quartz substrates. (d) Surface density distribution of pyrene on these substrates, with an average value of 0.251 ± 0.039 nmol/cm² (0.723 ± 0.028 g/m²).


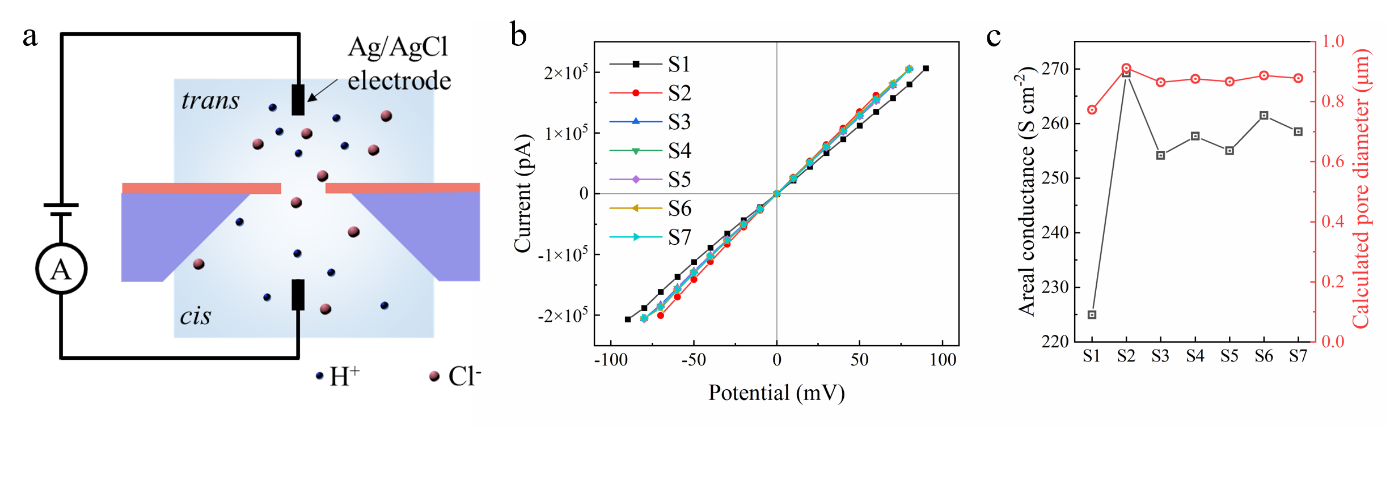
**Figure S4.** (a) Schematic of the ionic current measurement setup. (b) I-V curves generated for seven randomly selected bare chips were obtained by fitting their current traces to a single exponential decay function. (c) Corresponding conductance values and calculated hole diameters for these chips, with markers matching the color scheme in (b).


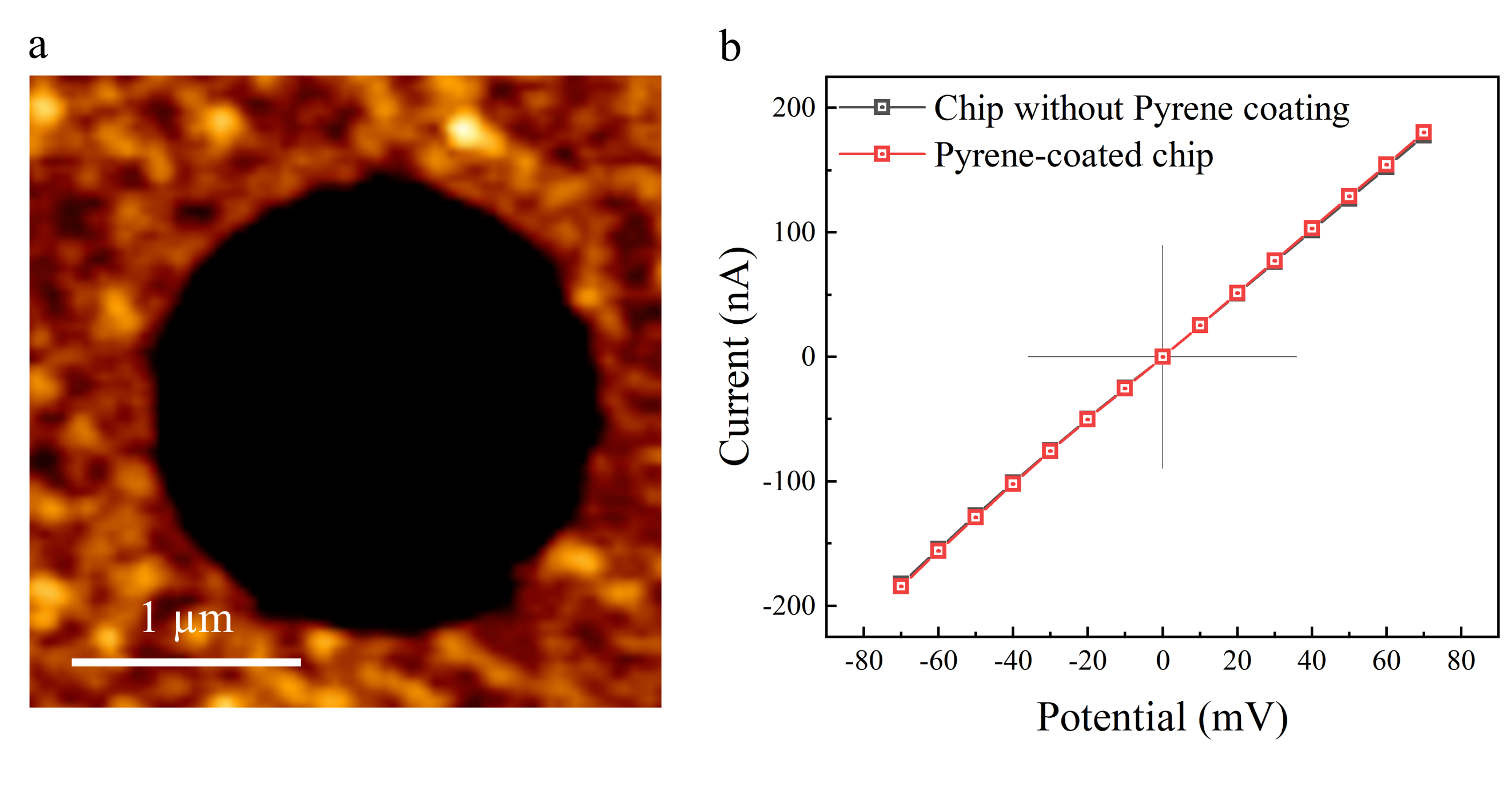


**Figure S5.** (a) Atomic force microscopy (AFM) image of the pyrene-coated chip, including the hole area. The 1 µm hole remains exposed, with no layer covering it. (b) I-V curves of the chip measured in 0.1 M HCl, comparing the conductance of the chip without a pyrene-coated layer and with a pyrene-coated layer.


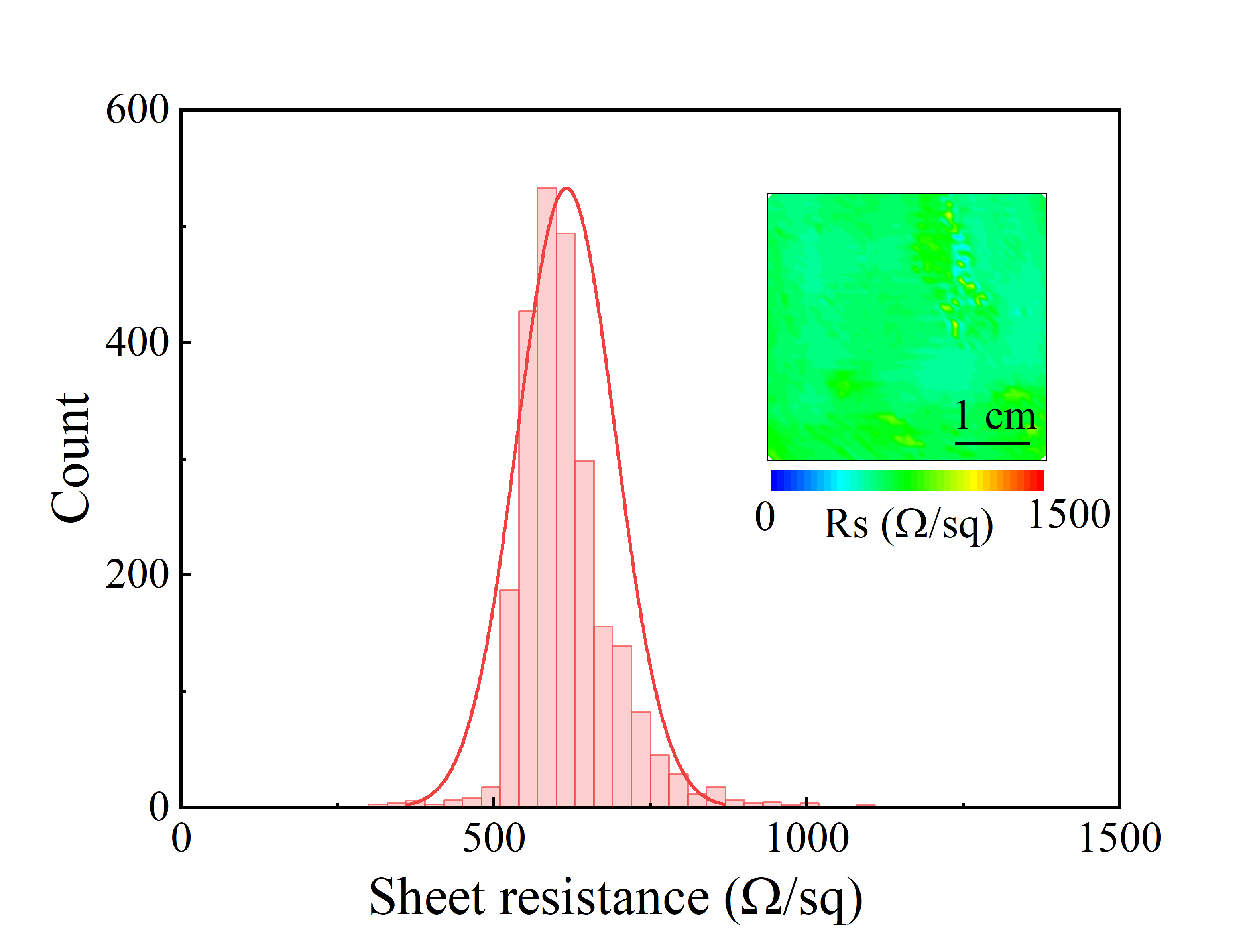


**Figure S6.** Sheet resistance measured across a 5 cm × 5 cm area, with the inset showing a mapping of the sheet resistance distribution.


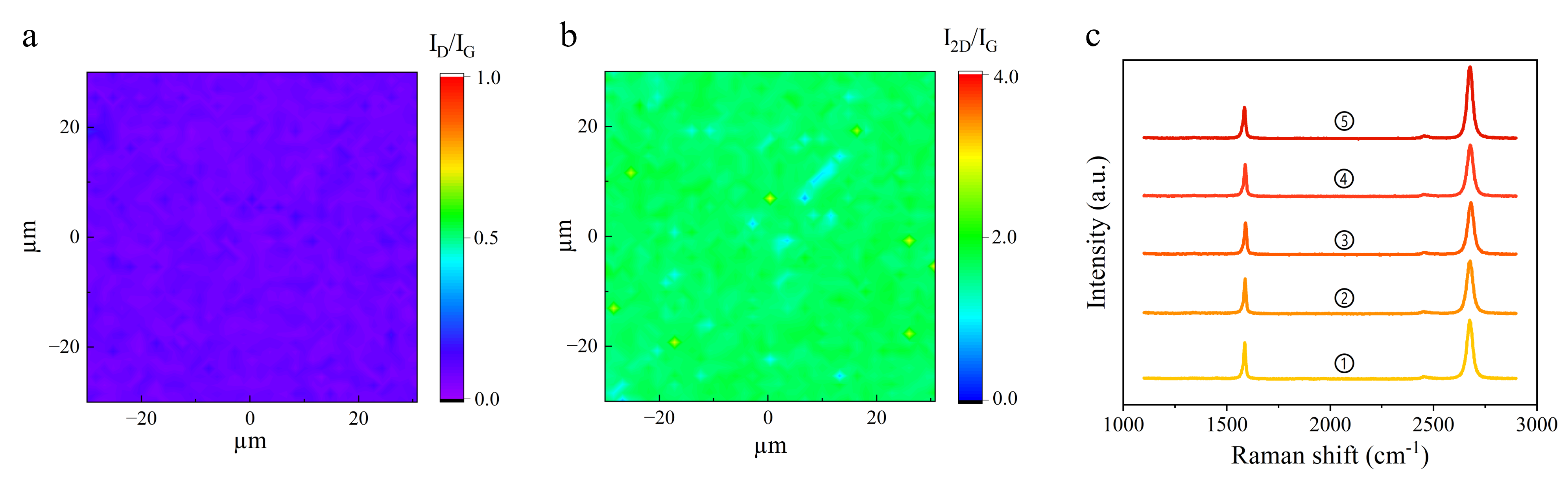


**Figure S7.** (a) Mapping of I_D_/I_G_ and (b) I_2D_/I_G_ ratios over a 60 µm × 60 µm area. (c) Raman spectra obtained from five different spots across a centimeter-scale area.


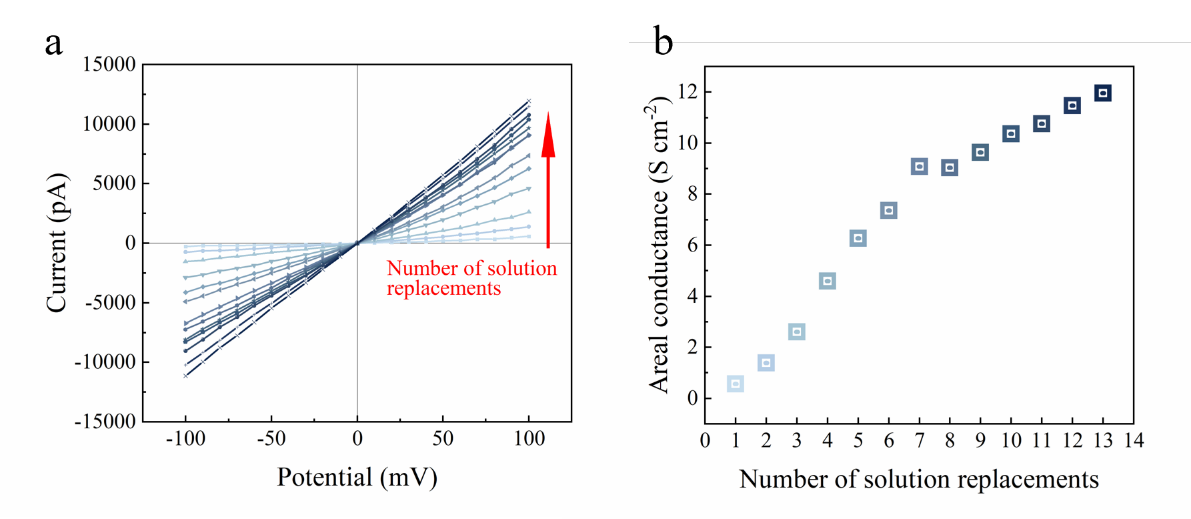


**Figure S8.** (a) I-V curves obtained from tests under different electrolyte exchange conditions in 0.1 M solution. (b) Areal conductance as a function of electrolyte replacement cycles.

**
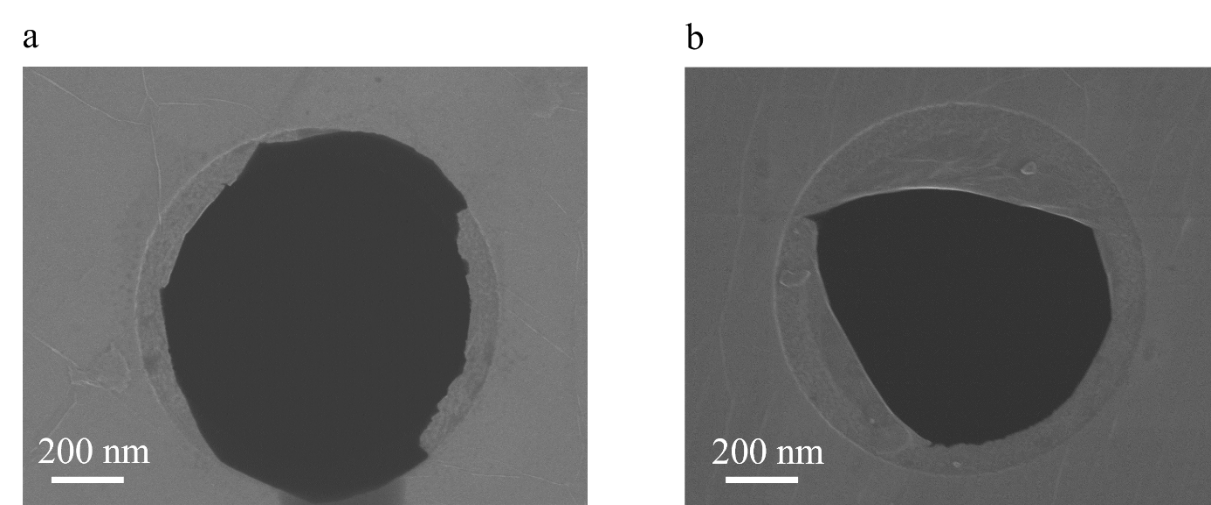
**

**Figure S9.** SEM images of graphene devices utilizing pyrene-modified chips, denoted as S15 and S16.

**
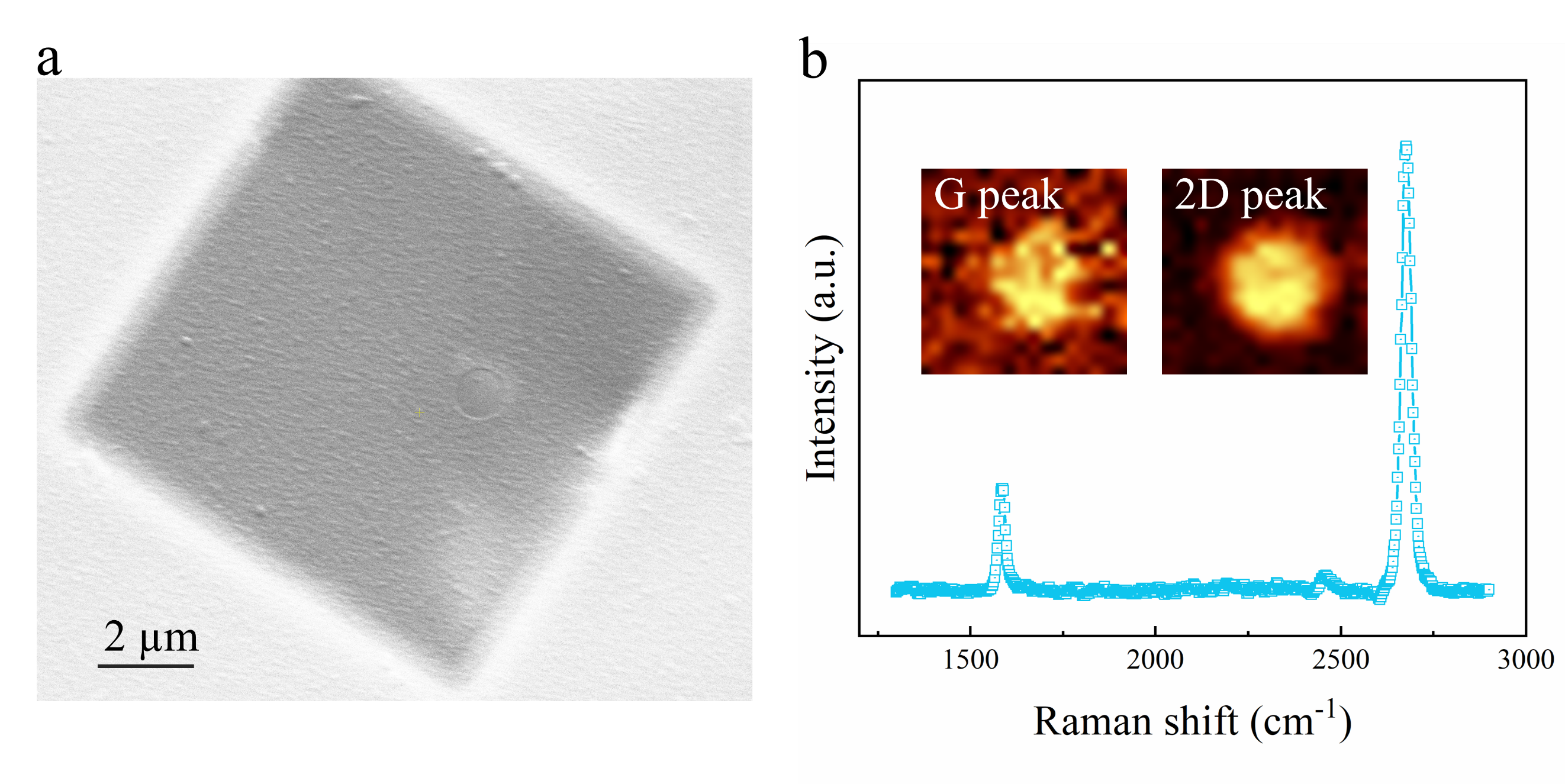
**

**Figure. S10.** (a) SEM image of silicon nitride (SiN) carrier chip with graphene after ion transport measurement. (b) Raman spectra of free-standing graphene area after ion transport measurement, with insets showing the mapping of the G peak and 2D peak.
